# Freezing of Vaginal Swabs Prior to DNA Purification Does Not Statistically Significantly Affect Microbiome Composition

**DOI:** 10.1002/mbo3.70053

**Published:** 2025-08-28

**Authors:** Khaled Saoud Ali Ghathian, Julie Elm Heintz, Sarah Mollerup, Sarah Juel Paulsen, Karen Angeliki Krogfelt, Niels Frimodt‐Møller, Katrine Hartung Hansen, Sofie Ingdam Halkjær, Anne Holm, Mette Pinholt, Andreas Munk Petersen

**Affiliations:** ^1^ Department of Clinical Microbiology Copenhagen University Hospital ‐ Amager and Hvidovre Hvidovre Denmark; ^2^ Department of Clinical Medicine, Graduate School of Health and Medical Sciences University of Copenhagen Copenhagen Denmark; ^3^ Department of Science and Environment, Molecular and Medical Biology, Center for Mathematical Modeling ‐ Human Health and Disease PandemiX Center Roskilde University Roskilde Denmark; ^4^ Department of Clinical Microbiology Copenhagen University Hospital ‐ Rigshospitalet Copenhagen Denmark; ^5^ Department of Clinical Medicine University of Copenhagen Copenhagen Denmark; ^6^ Gastrounit, Medical Section Copenhagen University Hospital ‐ Amager and Hvidovre Hvidovre Denmark; ^7^ Department of Public Health, Medicine Section University of Copenhagen Copenhagen Denmark

**Keywords:** DNA extraction, DNA purification, human DNA depletion, microbiome composition, vaginal microbiome

## Abstract

Vaginal microbiome profiles may be affected by storage conditions, especially after human DNA depletion, yet systematic evaluations are limited. To assess the short‐term impact of storage temperature on vaginal microbiome composition following host DNA depletion. Vaginal swabs were stored at 5°C (48 h), −20°C (3 weeks), or −80°C (3 weeks). DNA was extracted using the MolYsis Complete5 kit and profiled with MetaPhlAn4. Alpha and beta diversity metrics and differential abundance tests (CLR‐transformed, Kruskal–Wallis and Wilcoxon with FDR correction) were applied. Internal quality controls (IQCs) assessed reproducibility and storage bias. No significant differences were found in alpha diversity, relative abundances (Kruskal–Wallis *p* = 0.786), or global beta diversity (ANOSIM *R* = − 0.042, *p* = 0.937). PERMANOVA showed a trend (*F* = 3.51, *p* = 0.061), but ANCOM‐BC2 found no differentially abundant taxa. IQCs revealed variation in low‐abundance Gram‐negative species after freezing. Vaginal microbiome composition remained largely stable under short‐term freezing conditions (−20°C and −80°C), supporting their use in clinical and research workflows. Nonetheless, subtle shifts in low‐abundance or fragile taxa may occur and should be interpreted with caution in studies emphasizing microbial fine structure or biomarker discovery.

## Graphical abstract

The vaginal microbiome is vital to women's health. We assessed the effects of short‐term storage and human DNA depletion on microbial profiles. While overall composition remained stable, trends in beta diversity and internal controls suggest subtle effects on low‐abundance species, highlighting the need for robust analytical design.

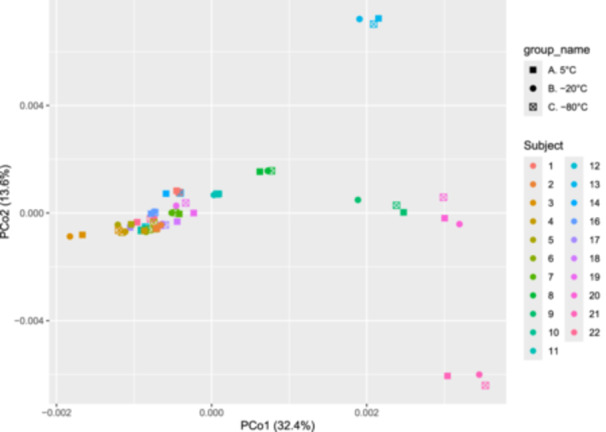

**Abbreviations**
ATCC: American Type Culture Collection
HD: human DNA depletion
IQC: internal quality control
McF: McFarland
PC: principal coordinate
PCoA: principal coordinate analysis

## 1 Introduction

The vaginal microbiome is gaining increased research interest due to proposed links between the composition of the vaginal microbiome and various aspects of female and reproductive health (France et al. 2022). Preanalytical variability of sample collection, transport, storage conditions, and general processing steps is crucial factors, when analyzing human clinical specimens with microbiological diagnostic tools (Sánchez‐Romero et al. 2019a; Allaband et al. 2019). Ideally, clinical samples should be processed as soon as possible after collection to minimize changes in the microbial composition. However, as immediate processing is often not possible, it is important to know how to store the samples to maintain the stability of the microbiome composition. Samples are typically stored at low temperatures to slow down microbial growth and prevent DNA degradation. The recommended temperature for short‐term storage (1–2 days) is usually around 2°C–8°C (Baron et al. 2013; Sánchez‐Romero et al. 2019b). For longer‐term storage, samples can be stored at −80°C to preserve the microbiome composition effectively (Li et al. 2023). However, freezing and thawing can lead to degradation of the microbes and their DNA, thereby affecting the accuracy of microbiome analysis (Poulsen et al. 2021). Only few studies have investigated the influence of temperature and storage conditions on microbiome analysis.

Metagenomic shotgun sequencing of human sample material can be challenged in tissues with a high ratio of human genomic material compared with microbial material. During sequencing, high amounts of human DNA simply decrease the sensitivity of microbial detection. Therefore, performing human DNA depletion (HD) as part of the DNA purification process may be advantageous, but may also impact the microbiome composition (Marotz et al. 2018). This study aimed to investigate the impact of storage conditions (temperature and duration) on the number of microbial reads, the efficiency of HD, and, not least, the microbiome composition of vaginal swab samples.

## 2 Methods and Materials

According to the procedure at the Department of Clinical Microbiology, Copenhagen University Hospital Hvidovre, Denmark, it is recommended that microbial DNA purification from human clinical samples for microbiome analyses must be carried out within 48 h from the time of collection, and the samples are stored at 5°C–8°C (COPAN Diagnostics 2024). In this study, we used the Copan ESwab (Copan, Italy) (COPAN Diagnostics 2024), a widely adopted clinical sample collection and transport system that is standard practice in our Department of Clinical Microbiology. This swab is validated for microbial viability for up to 48 h in room or refrigerated conditions, which aligns with our typical sample handling and processing timeline. The MolYsis Complete5 DNA extraction kit (Molzym GmbH & Co. KG, Bremen, Germany) used for microbial DNA purification recommends that the procedure must be performed on fresh human samples (Molzym GmbH & Co. KG 2024). However, due to logistical reasons, it is not always possible to perform microbial DNA purification within 48 h. By investigating the effects of storage parameters such as temperature, duration, and preservatives, we can optimize sample handling protocols to ensure the integrity of microbiome data. The protocol for this study was designed as follows.

### 2.1 Study Population and Sample Collection

Twenty‐one healthy women, 18–40 years of age, were recruited for this study. Vaginal swab samples were self‐collected by each participant using Copan 1 mL Liquid Amies Elution Swab (ESwab) (Copan, Italy) as recommended by the manufacturer to minimize variability. The instructions aimed to ensure that samples were collected from the midvaginal wall (approximately 5 cm and rotated carefully for approximately 30 s) to obtain representative microbiome profiles. Samples were initially stored at 5°C. Within 48 h each sample was divided into three subsamples and stored at either (Group A) 5°C for maximum 48 h, (Group B) −20°C for 3 weeks, or (Group C) −80°C for 3 weeks, respectively. Samples in Groups B and C were mixed 1:1 with Müller–Hinton broth containing 10% glycerol before freezing, with a final concentration of 5% glycerol.

### 2.2 DNA Purification and Sequencing

Microbial DNA purification was performed with the MolYsis Complete5 DNA extraction kit (Molzym GmbH & Co. KG, Bremen, Germany) (Molzym GmbH & Co. KG 2024). The method includes pretreatment of samples to remove human DNA. Samples in Group A were processed for microbial DNA purification within 48 h of sampling. Samples in Groups B and C were stored at −20°C or −80°C for 3 weeks before DNA purification. DNA purification was performed using the MolYsis Complete5 kit according to the manufacturer's instructions with the following adjustments: lysis of microbes was performed using a thermoshaker at 250 rpm for 30 min at 37°C, no ß‐mercaptoethanol was used in this step. Next Proteinase K treatment was performed in a heating block at 56°C for 10 min (samples were shortly vortexed after 3 and 7 min). DNA concentration was measured on a Qubit 2.0 Fluorometer quantification (Invitrogen, Life Technologies, CA 92008, USA) using the Qubit 1X double‐stranded DNA HS Assay Kit (Invitrogen, Cat. Nr. Q33231, Eugene, Oregon, USA). Subsequently, DNA libraries were prepared on a Biomek 4000 (Bechman Coulter, Indianapolis, USA) with Nextera XT DNA Sample Preparation Kit (Illumina Inc., San Diego, CA, USA) according to the manufacturer's instructions. Shotgun sequencing was performed on a MiSeq instrument (Illumina Inc., San Diego, USA) using MiSeq Reagent Kit v2 (300 cycle) (Illumina Inc., Cat. Nr. 15033412), generating 2 × 150 bp paired‐end reads. This study employed shotgun metagenomic sequencing. DNA was enzymatically fragmented during library preparation using the Nextera XT DNA Sample Preparation Kit (Illumina Inc.), which combines tagmentation and adapter ligation. Fragmentation was validated indirectly through successful library yield and quality control metrics during sequencing.

### 2.3 Bioinformatic Analysis

Raw shotgun metagenome reads were trimmed using fastp v. 0.20.1 (Chen et al. 2018) with qualified_quality_phred of 20 and a minimum read length of 50. FastQC v. 0.11.8 (Andrews 2010) was used for evaluating the quality of the reads before and after trimming. Removal of human sequencing reads was performed by aligning the trimmed reads to the human genome (hg38, University of California, Santa Cruz) using bowtie2 v. 2.3.4.1 (Langmead and Salzberg 2012) with end‐to‐end alignment and maximum fragment length for valid paired‐end alignments (‐X) of 2000. Clade‐based microbial profiling of the human depleted reads was performed with MetaPhlAn v. 4.1.1 (Blanco‐Míguez et al. 2023) (database version mpa_vJun23_CHOCOPhlAnSGB_202403) with the addition of the parameters ignore_eukaryotes, ignore_usgbs, and t rel_ab_w_read_stats.

### 2.4 Taxonomic Analysis

The taxonomic data was processed using R v. 4.3.0 (Team RC. 2021) in RStudio v. 2023.06.0 (Posit 2023) and ggplot2 v. 3.5.0 (Create Elegant Data Visualisations Using the Grammar of Graphics · ggplot2 Internet 2024) was used for visualizations. Beta diversity analysis was performed using QIIME2 v. 2023.09 (Bolyen et al. 2019) using the species‐level estimated read counts generated by MetaPhlAn4. Aitchison distance was used as a beta diversity metric to account for the compositionality of the data (Galloway‐Pena et al. 2017) and was calculated using the QIIME2 diversity plugin, adding a pseudocount of 1. Principal coordinate analysis (PCoA) was calculated using the ecodist R‐package v. 2.0.9 (Goslee and Urban 2007).

### 2.5 Internal Quality Control (IQC)

**Figure 1:**
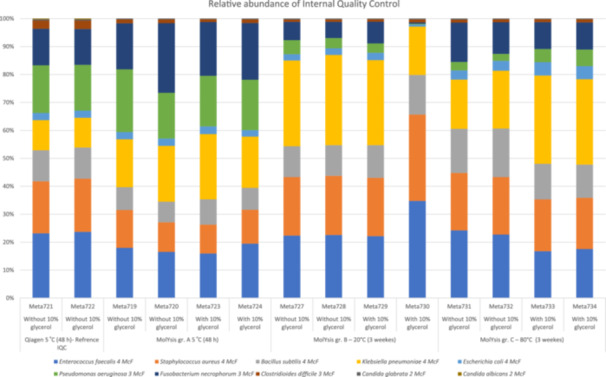
Internal quality control (IQC). Relative abundance of DNA reads (%) of the IQC was analyzed using the same protocol as for vaginal swabs, except sample Meta 721 and Meta 722 (used as reference IQC value tested by Qiagens DNeasy PowerSoil Pro Kit—without human DNA depletion). Suspension of a mixture of ATCC strains: *Escherichia coli* ATCC 25922, *Klebsiella pneumoniae* ATCC 700603, *Pseudomonas aeruginosa* ATCC 27853, *Fusobacterium necrophorum* ATCC 25286, *Enterococcus faecalis* ATCC 29212, *Staphylococcus aureus* ATCC 25923, *Bacillus subtilis* ATCC 6051, *Clostridium difficile* ATCC 43255, *Candida albicans* ATCC 90028, and *Candida glabrata* ATCC 90030. The suspension mixture was stored at different conditions: Groups A (5°C, 48 h), B (−20°C, 3 weeks), and C (−80°C, 3 weeks) with and to without 10% glycerol (dilution 1:1). The reference IQC tested by Qiagen was kept at 5°C and analyzed within 48 h. ATCC, American Type Culture Collection.

We developed an IQC composed of 10 strains (four Gram‐negative bacteria, four Gram‐positive bacteria, and two Candida species) obtained from the American Type Culture Collection (ATCC) (Figure 1). This IQC setup allowed us to evaluate the robustness and reproducibility of the HD and microbial DNA extraction processes under varying storage conditions. The microorganisms were cultured on agar plates, and after 24 h of incubation, they were individually suspended in 0.9% saline solution. The concentrations of the suspensions were determined using McFarland standards (McF) measured with the DensiCHEK Plus system (BioMérieux S.A., Marcy l'Étoile, France), 0.5 McF corresponds approximate Cell Density 1.5 × 10^8^/mL (Donay et al. 2007). The concentrations were adjusted as follows: *Klebsiella pneumoniae* at 4 McF, *Escherichia coli* at 4 McF, *Pseudomonas aeruginosa* at 3 McF, *Fusobacterium necrophorum* at 3 McF, *Enterococcus faecalis* at 4 McF, *Staphylococcus aureus* at 4 McF, *Bacillus subtilis* at 4 McF, *Clostridium difficile* at 3 McF, *Candida glabrata* at 2 McF, and *Candida albicans* at 2 McF.

The 10 dilutions were pooled and then subdivided into 14 samples. Six samples were mixed in a 1:1 ratio with Müller–Hinton broth containing 10% glycerol, and six samples remained without the broth–glycerol mixture. The samples were stored under three conditions: four samples (to with and without 10% glycerol—dilution 1:1) at 5°C for 48 h, four at −20°C for 3 weeks, and four at −80°C for 3 weeks. All IQC samples were processed and analyzed in duplicate using the same protocol employed for vaginal swabs. To obtain a baseline measure (IQC reference value), we purified the two IQC samples without performing HD, using Qiagens DNeasy PowerSoil Pro Kit (QIAGEN GmbH, Germany) (QIAGEN 2024). The reference IQC samples tested by Qiagen were kept at 5°C and analyzed within 48 h.

#### 2.5.1 Statistical Analysis

The obtained microbial data were analyzed to compare the composition and diversity of the vaginal microbiome under different storage conditions. Statistical methods, including multivariate analysis, were employed to identify significant differences by looking at total reads, bacterial reads, species counts, nonhumans fraction and variation in species composition between different subjects and groups.

Data were analyzed by using DATAtab web‐based statistics software (DATAtab: Online Statistics Calculator 2024) and Analysis of Composition of Microbiomes with Bias Correction (ANCOM‐BC_2.4.0). Normal distribution tests (Kolmogorov–Smirnov, Kolmogorov–Smirnov [Lilliefors Corr.], Shapiro–Wilk and Anderson–Darling tests and Histogram Graphical and a quantile‐quantile plot [Q–Q plot]) were used to evaluate whether to use parametric or nonparametric statistical tests. Second, differences between the three groups were investigated with One‐factorial Analysis of Variance (One‐way ANOVA) for normally distributed data, and the Kruskal–Wallis test for nonnormally distributed data. A *p* value smaller than 0.05 was considered statistically significant for both types of tests. The analysis of similarity (ANOSIM) test is used to test if there is a statistical difference between the microbial communities of two or more groups of samples. Diversity metrics (Shannon, Simpson, Bray–Curtis, Jaccard) and differential abundance (centered log‐ratio [CLR]–transformed, Kruskal–Wallis and Wilcoxon tests with Benjamini–Hochberg correction) were used for analysis. A *q* < 0.05 was considered statistically significant to account for multiple testing and control the false discovery rate (FDR).

## 3 Results

To validate microbial purification, we measured and compared the average total DNA concentration after purification. The measured average concentrations of total DNA (microbial and nonmicrobial) after purification across the categories were 6.12 ng/µL in Group A, 10.58 ng/µL in Group B, and 11.63 ng/µL in Group C. The number of bacterial species was 174 in Group A, 153 in Group B, and 146 in Group C.

### 3.1 Internal Quality Control

As we have not been able to identify a commercially available microbiome quality control sample suitable for DNA purification, including HD, we designed an IQC (Figure 1). To obtain a baseline measure and reference value, we purified the IQC without performing HD, using Qiagens DNeasy PowerSoil Pro Kit. The analysis of our IQC revealed that the DNA extraction process utilizing the MolYsis Complete5 DNA extraction kit did have an influence on the microbiome composition, and that freezing exacerbated this (Figure 1). However, even though we saw differences in the bacterial distribution, we were able to detect all bacteria as only *C. albicans* was undetectable. The most prominent changes in the abundance of reads mount were on Gram‐negative bacteria, *E. coli* and *P. aeruginosa*. In the IQC samples, we observed a decrease in the relative abundance of *P. aeruginosa* and *F. necrophorum* specifically in the −20°C (3 weeks) condition, alongside a relative increase in *K. pneumoniae* (Figure 1). However, both *P. aeruginosa* and *F. necrophorum* were among the lower‐abundance spiked‐ins and showed greater interreplicate variability, making it difficult to distinguish true biological effects from technical variation. In contrast, *K. pneumoniae* had higher overall abundance and more consistent detection across conditions.

### 3.2 Differential Abundance Analysis Across Storage Temperatures

To investigate the effect of storage temperature on microbial composition, we conducted differential abundance testing using analysis of composition of microbiomes with bias correction 2 (ANCOM‐BC2) at both the family and species levels. Using the updated analysis, we did not find any taxa that were significantly differentially abundant across the three temperature conditions (5°C, −20°C, and −80°C). At the species level, no taxa met the threshold for statistical significance (*q* < 0.05), and all tested species exhibited *q* values above 0.1. Similarly, at the family level, none of the taxa displayed significant changes across temperature treatments; the lowest observed *q* value was for *Peptoniphilaceae* (*q* = 0.161), which did not reach significance. These results suggest that microbial composition remains relatively stable across different storage temperatures at both the family and species levels.

### 3.3 Microbial Diversity Across Storage Temperatures

**Figure 2:**
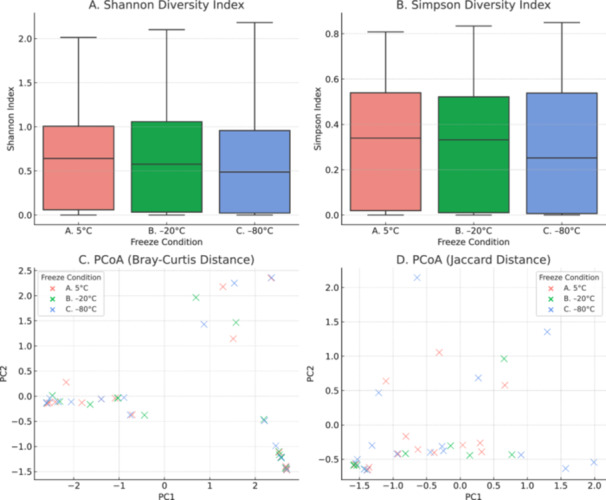
Alpha and beta diversity analyses of vaginal microbiota across different freezing conditions. (A) Shannon and (B) Simpson diversity indices indicate no significant differences in microbial richness or evenness between samples stored at 5°C for 48 h (Group A, 5°C), −20°C for 3 weeks (Group B, −20°C), or −80°C for 3 weeks (Group C, −80°C). (C, D) Principal Coordinate Analysis (PCoA) based on Bray–Curtis (C) and Jaccard (D) distances shows no clustering by freeze condition, suggesting a negligible effect of storage temperature on overall bacterial community composition. Statistical testing using ANOSIM showed no significant group differences (*R* = − 0.042, *p* = 0.937). ANOSIM, analysis of similarity; PC, principal coordinate.

Figures 2 and 4 illustrate the effect of different storage temperatures (5°C, −20°C, and −80°C) on microbial diversity using both alpha and beta diversity metrics. Panels A and B show the Shannon and Simpson diversity indices, respectively. Across the three temperature conditions, there were no significant differences in alpha diversity (Figures 2 and 4). This indicates that species richness and evenness were largely maintained regardless of storage temperature. Panels C and D present PCoA based on Bray–Curtis and Jaccard distance metrics. These analyses also showed no distinct clustering based on storage temperature. Instead, microbial community structure appeared consistent across conditions, suggesting that overall beta diversity remained stable (Figures 2 and 4). Collectively, these results indicate that short‐term storage at 5°C, −20°C, or −80°C does not substantially alter microbial diversity, supporting the robustness of microbial community assessments across these storage treatments.

### 3.4 Differential Abundance Analysis Across Freezing Conditions

To assess whether specific bacterial taxa were differentially abundant between freezing treatments, we applied a nonparametric statistical pipeline appropriate for compositional data. After filtering out low‐abundance species (mean relative abundance < 0.01%), data were transformed using the CLR method. A global Kruskal–Wallis test across the three groups (Group A, 5°C for 48 h; Group B, −20°C for 3 weeks; Group C, −80°C for 3 weeks) revealed no statistically significant differences in species‐level abundance (FDR‐adjusted *q* > 0.98 for all taxa, adjusted using the Benjamini–Hochberg method, Hu et al. 2018).

**Figure 3:**
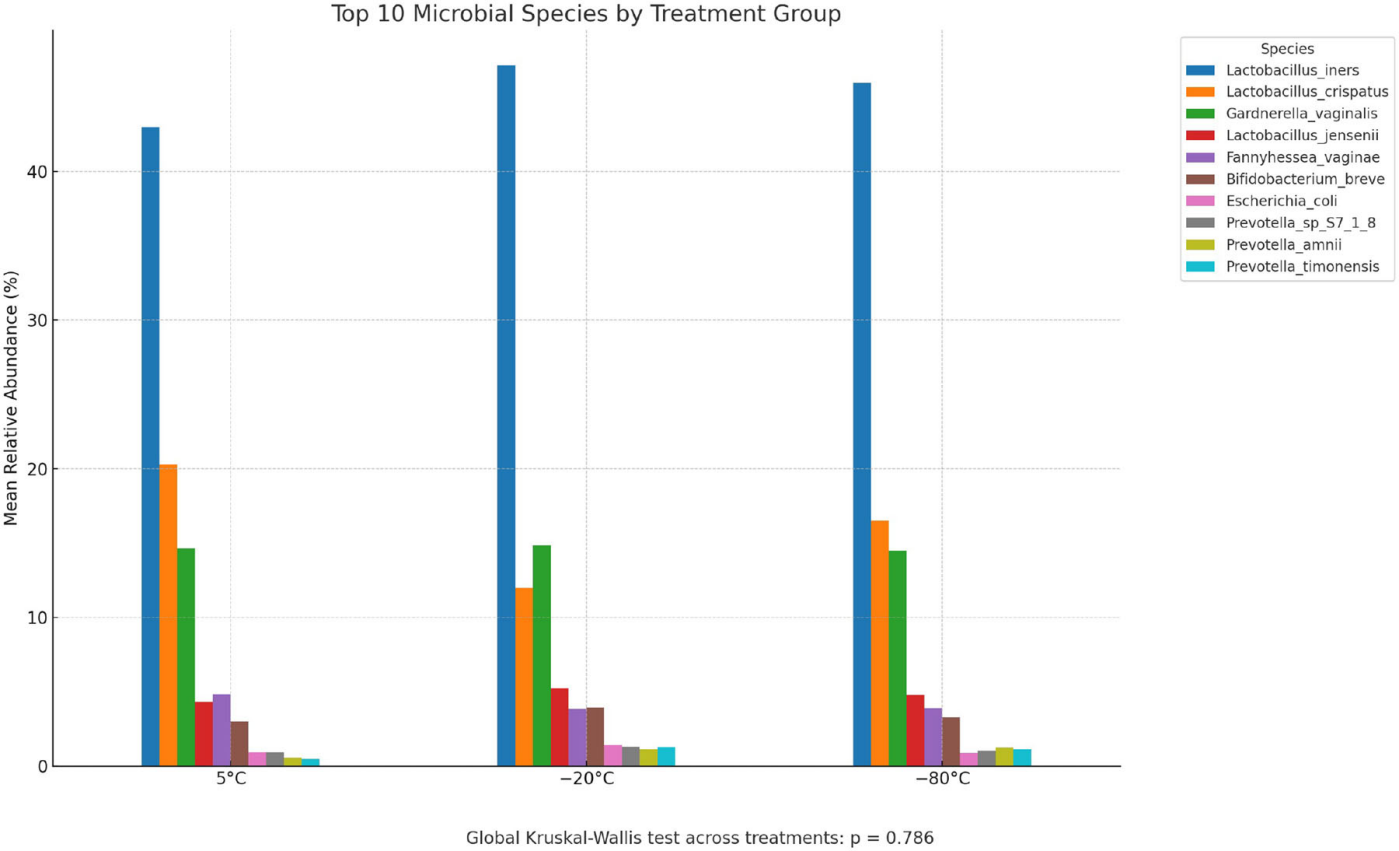
Relative abundance of the top 10 microbial species. Relative abundance of the top 10 microbial species grouped by treatment condition (5°C, −20°C, and −80°C). Each bar represents the mean relative abundance of a species across all samples in that group. A global Kruskal–Wallis test comparing microbial composition across the three treatments yielded no statistically significant differences (*p* = 0.786).

Subsequent pairwise Wilcoxon rank‐sum tests also identified no significant differences between any group pairs (all FDR‐adjusted *q* > 0.91, adjusted using the Benjamini–Hochberg method). These findings suggest that the relative abundance of dominant bacterial taxa remains stable across short‐term freezing conditions. A summary of the relative abundances of the top 10 bacterial species by treatment group is presented in Figure 3. Analysis of the relative abundance of the top 10 bacterial species across storage conditions (Figure 3) revealed no statistically significant differences (global Kruskal–Wallis *p* = 0.786). This suggests that dominant species remained stable regardless of storage at 5°C, −20°C, or −80°C. These results are consistent with the findings from alpha diversity and ANOSIM analyses, further supporting the overall resilience of the vaginal microbiome to short‐term freeze storage.

### 3.5 Beta Diversity

**Figure 4:**
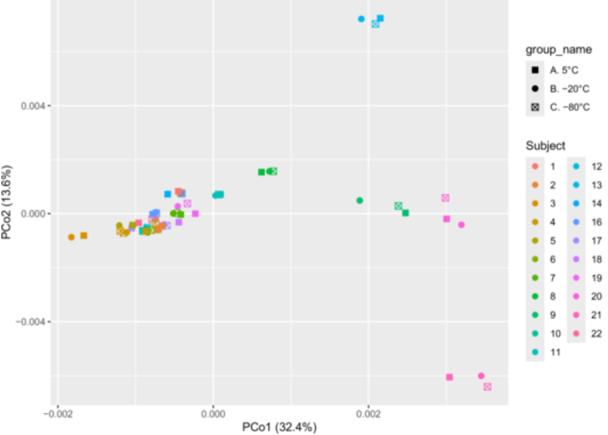
Beta diversity test. Beta diversity of the variation in bacterial species composition across vaginal swab samples under three storage conditions: Group A (5°C, 48 h), Group B (−20°C, 3 weeks), and Group C (−80°C, 3 weeks). The figure shows a Principal Coordinate Analysis (PCoA) plot based on Bray–Curtis dissimilarity, visualizing differences in microbial communities among the three groups (*n* = 21 samples), with samples color‐coded by group. The first two principal coordinates explain 32.4% and 13.6% of the total variance, respectively. Statistical differences in beta diversity among groups were tested using an ANOVA‐based approximation of PERMANOVA, revealing a trend toward significance (*F* = 3.51, *p* = 0.061), suggesting potential group‐level differences in community composition. ANOVA, analysis of variance; PERMANOVA, permutational multivariate analysis of variance.

Analysis of alpha diversity revealed no significant differences in microbial richness or evenness among the three storage groups (5°C for 48 h, −20°C for 3 weeks, −80°C for 3 weeks), as measured by Shannon and Simpson diversity indices (Figure 2A,B). Likewise, PCoA based on Bray–Curtis and Jaccard distances showed no visible clustering of samples by freeze condition (Figure 2C,D). Statistical testing using ANOSIM confirmed a lack of significant group differences (*R* = − 0.042, *p* = 0.937). In contrast, a separate beta diversity (Figure 4) analysis using an ANOVA‐based approximation of permutational multivariate analysis of variance (PERMANOVA) on Bray–Curtis distances revealed a trend toward significance (*F* = 3.51, *p* = 0.061), suggesting possible group‐level differences in microbial community structure (Figure 4). However, this finding was not statistically significant at the conventional threshold (*p* < 0.05).

### 3.6 Total Trimmed Reads

Following sequencing, raw sequencing data undergo a quality control process known as “trimming.” During trimming, low‐quality bases, adapters, and other unwanted sequences are removed from the raw reads, resulting in cleaner and more accurate sequences. Supporting Figure 1 shows the total trimmed reads for Groups A–C. The total trimmed reads were highest in sample Group B, but no significant difference in total trimmed reads was found between the three groups, *p* = 0.297 (Table 1). Supporting Figure 1 shows that the average of total trimmed reads was approximately 809,225.14 for Group A, 1,070,537.81 for Group B, and 956,528.48 for Group C. The overall average, considering all groups, is 945,430.48.

**Table 1:** Normal distribution test between the groups for total trimmed reads.

	Total trimmed reads		
	Group A, 5°C	Group B, −20°C	Group C, −80°C
*Normal distribution*			
Kolmogorov–Smirnov	*p* = 0.849	*p* = 0.571	*p* = 0.945
Kolmogorov–Smirnov (Lilliefors Corr.)	*p* = 0.521	*p* = 0.148	*p* = 0.820
Shapiro–Wilk	*p* = 0.666	*p* = 0.051	*p* = 0.346
Anderson–Darling	*p* = 0.541	*p* = 0.084	*p* = 0.478
*ANOVA test*	*p* = 0.297		

*Note:* Data analysis of variance showed that there was no significant difference between Groups A–C, *p* = 0.297.

### 3.7 Bacterial Reads and Relative Abundance

Supporting Figure 2 shows the number of reads classified as bacterial by MetaPhlAn for Groups A–C, and there was no significant difference concerning bacterial reads between the three groups *p* = 0.09 (Table 2). Supporting Figure 3 shows the relative abundance of the bacterial species detected in the samples in the three sample groups. The number of bacterial species identified in Groups A–C, were not significantly different among the three groups, *p* = 0.726 (Table 3). The highest number of bacterial species was found in Group A (*n* = 174), compared with Groups B and C, which had 153 and 146 species, respectively.

**Table 2:** Normal distribution test between the groups for bacterial reads.

	Bacterial reads		
	Group A, 5°C	Group B, −20°C	Group C, −80°C
*Normal distribution*			
Kolmogorov–Smirnov	*p* = 0.423	*p* = 0.058	*p* = 0.148
Kolmogorov–Smirnov (Lilliefors Corr.)	*p* = 0.061	*p* < 0.001	*p* = 0.003
Shapiro–Wilk	*p* = 0.012	*p* < 0.001	*p* < 0.001
Anderson–Darling	*p* = 0.011	*p* < 0.001	*p* < 0.001
*Kruskal–Wallis test*	*p* = 0.09		

*Note:* Data analysis of variance showed that there was no significant difference for bacterial reads between the three groups A–C, *p* = 0.09.

**Table 3:** Normal distribution test between the groups for species count.

	Species count		
	Group A, 5°C	Group B, −20°C	Group C, −80°C
*Normal distribution*			
Kolmogorov–Smirnov	*p* = 0.029	*p* = 0.072	*p* = 0.032
Kolmogorov–Smirnov (Lilliefors Corr.)	*p* < 0.001	*p* < 0.001	*p* < 0.001
Shapiro–Wilk	*p* < 0.001	*p* < 0.001	*p* < 0.001
Anderson–Darling	*p* < 0.001	*p* < 0.001	*p* < 0.001
*Kruskal–Wallis test*	*p* = 0.726		

*Note:* The results of the different tests are contradictory, and therefore, the species count is not normally distributed. There is no significant difference in species count between the three groups A–C, *p* = 0.726.

Examining the identified bacteria species further, we investigated “undetected bacteria,” where an undetected bacterium refers to one that is found in one group but not in the other groups. The comparison of the groups in terms of undetected bacterial genera reveals that 17 genera were absent in Group A, whereas Groups B and C had 36 and 45 undetected bacterial genera, respectively. Table 4 shows 62 undetected bacterial genera from all three groups were almost all anaerobic bacteria distributed as follows: the most abundant anaerobic genera, that we could not detect across the three groups were *Anaerococcus* genus (*n* = 14) at Group A (*n* = 1), Group B (*n* = 6), and Group C (*n* = 7), *Prevotella* genus (*n* = 14) was distributed across the three different groups A, B, and C as follows: 4, 7, and 3, respectively. *Peptoniphilus* genus (*n* = 12) was distributed across the three different groups A, B, and C as follows: 1, 5, and 6, respectively, and 22 other anaerobic genera. Considering which types of genera are most often undetected, we observed that anaerobic bacteria make up the largest proportion at 63.3% (*n* = 62), facultative anaerobic bacteria constitute 12.2% (*n* = 12), and aerobic bacteria account for 24.5% (*n* = 24).

**Table 4:** Overview of nondetected anaerobic bacteria.

Undetected anaerobic bacteria	Group A, 5°C	Group B, −20°C	Group C, −80°C
*Anaerococcus vaginalis*	0	1	2
*Anaerococcus murdochii*	1	1	2
*Anaerococcus tetradius*	0	1	0
*Anaerococcus lactolyticus*	0	1	1
*Anaerococcus hydrogenalis*	0	2	2
*Prevotella bivia*	1	0	0
*Prevotella timonensis*	0	2	2
*Prevotella buccalis*	2	3	1
*Prevotella colorans*	0	1	0
*Prevotella corporis*	1	1	0
*Peptoniphilus coxii*	0	3	4
*Peptoniphilus lacrimalis*	0	0	1
*Peptoniphilus duerdenii*	1	2	1
*Other anaerobic bacteria*	3	8	11
Total	9	26	27

*Note:* This table shows the number of undetected anaerobic bacteria across the three groups. The three genera *Anaerococcus, Prevotella*, and *Peptoniphilus* are affected by the microbial DNA purification process and storage conditions at −20°C to −80°C, respectively.

### 3.8 Nonhuman Fraction of Trimmed Reads

**Figure 5:**
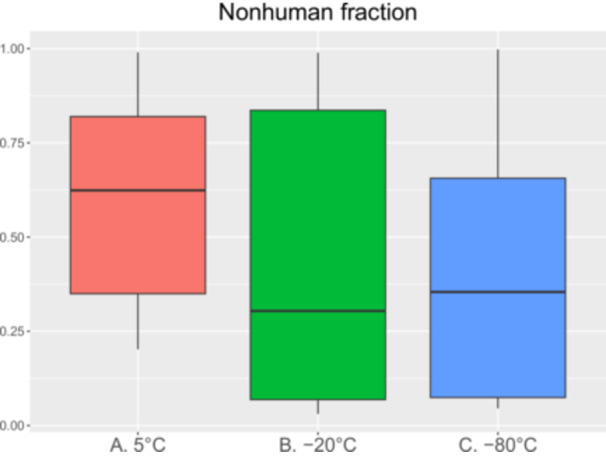
Overview of nonhuman fraction. Nonhuman fraction of trimmed reads—the genetic material that comes from the microbiota and derived from the human host sample, from the different groups A (5°C, 48 h), B (−20°C, 3 weeks), and C (−80°C, 3 weeks).

Figure 5 shows the nonhuman fraction of trimmed reads between the groups and the effectiveness of HD. Figure 5 shows that the median of Group A is 0.62, while for Groups B and C, the medians are 0.3 and 0.35, respectively. This could imply that the freezing process influences the quality of HD negatively, where the risk of a higher amount of human DNA is greatest with freezing and storing for a longer period of 3 weeks compared with samples being analyzed after storage at 5°C within 48 h. There is evidence suggesting that freezing may increase the risk of moving microbial DNA during the HD step of the purification process. However, there was no significant difference of nonhuman fraction of reads between the three groups A–C, *p* = 0.186 (Table 5).

**Table 5:** Normal distribution test between the groups for the nonhuman fraction of trimmed reads.

	Nonhuman fraction of trimmed reads		
	Group A, 5°C	Group B, −20°C	Group C, −80°C
*Normal distribution*			
Kolmogorov–Smirnov	*p* = 0.628	*p* = 0.346	*p* = 0.663
Kolmogorov–Smirnov (Lilliefors Corr.)	*p* = 0.197	*p* = 0.033	*p* = 0.233
Shapiro–Wilk	*p* = 0.132	*p* = 0.003	*p* = 0.022
Anderson–Darling	*p* = 0.257	*p* = 0.003	*p* = 0.043
*Kruskal–Wallis test*	*p* = 0.186		

*Note:* Table 5 shows that the nonhuman fraction of trimmed reads is not normally distributed since the results of the different tests are contradictory. Data shows that there is no significant difference between the three groups A–C, *p* = 0.186.

## 4 Discussion

This study investigated the impact of temperature conditions and storage conditions on the isolation of microbial DNA for analysis of microbiome composition in vaginal swabs. Various parameters, including the number of total trimmed reads, bacterial reads, nonhuman fraction of trimmed reads, detected bacterial species, and diversity of the variation in species composition, did not differ significantly at any conditions. These findings are consistent with a study by Ahannach et al. (2021), where vaginal swabs were analyzed using 16S ribosomal RNA gene amplicon sequencing and showed an almost identical taxonomic composition across different storage conditions. They stored the first set of vaginal swabs at 4°C and processed them within 3 h after sampling (T1). All other samples were stored for 3 weeks at 4°C, followed by 3 days at room temperature. The second set was processed immediately (T2), while the third set was frozen for 3 days at −20°C and then processed (T3). The last set underwent host DNA depletion at T2 and, after 3 days at −80°C, DNA extraction (T4).

Our ANCOM‐BC2 analysis did not identify any taxa as significantly differentially abundant across storage temperatures, either at the family or species level. These results indicate that the microbial community structure remains relatively stable under the tested storage conditions (5°C, −20°C, and −80°C), reinforcing the reliability of these preservation methods for microbiome analyses. Even though we observed no statistically significant changes, the use of high‐resolution taxonomic and compositional analysis remains important in studies of storage‐related bias.

Previous research supports the robustness of microbial profiles under freezing conditions. Fouhy et al. (2015) found no significant differences at the phylum or family level in fecal microbiota when comparing fresh, snap‐frozen, and −80°C frozen samples using both MiSeq sequencing and culture‐based methods. Minor differences were noted only at the genus level, and interindividual variation was found to be more influential than storage condition. Culture‐based results also showed that levels of aerobes, anaerobes, and bifidobacteria were consistent across fresh and frozen conditions, further supporting the resilience of microbiota to short‐term freezing.

Similarly, Dominianni et al. (2014) evaluated various fecal sample collection and storage methods, including room temperature storage, RNAlater, and immediate −80°C freezing over a 3‐day period. They reported no significant differences in overall microbial community structure or in the relative abundance of key phyla (Firmicutes, Bacteroidetes, and Actinobacteria) across these methods. Their analysis demonstrated that microbiome profiles were more strongly influenced by interindividual differences than by the method of collection or storage. They also observed that DNA purity was somewhat lower when RNAlater was used, which may affect downstream applications, such as PCR.

Taken together, these findings, along with our results, suggest that microbial community profiles are generally stable under various short‐term storage conditions, including freezing and even room temperature, when samples are protected appropriately. This supports the feasibility of using these methods in large‐scale studies where immediate processing may not be practical.

While no taxa met the significance threshold in our analysis, the potential for subtle effects remains, especially at the genus or species level and in low‐abundance taxa. As demonstrated in both cited studies, such subtle variations may be undetectable without sufficient statistical power or sequencing depth. Therefore, the application of rigorous statistical models like ANCOM‐BC2 is essential, as they correct for compositionality and sequencing depth, thereby improving sensitivity in detecting true biological differences.

The absence of significant differences among the top 10 most abundant species across treatment groups further suggests that dominant members of the vaginal microbiome may be stable under the tested storage conditions. This aligns with previous studies showing that storage conditions have a limited impact on the structure of dominant microbial communities. For instance, Lauber et al. (2010) found that short‐term storage at temperatures ranging from 20°C to −80°C did not significantly affect the phylogenetic composition or diversity of microbial communities from human feces, skin, or soil, with interpersonal variation outweighing storage effects. Similarly, Choo et al. (2015) reported that refrigeration at 4°C preserved fecal microbial diversity and composition comparably to −80°C freezing, whereas room temperature storage or certain preservatives introduced compositional shifts, particularly in low‐abundance taxa such as *Anaerostipes* and *Bifidobacterium*.

While no individual taxa reached statistical significance in our ANCOM‐BC2 analysis, integrating both global diversity metrics and taxon‐level statistical tools remains critical for detecting subtle or taxon‐specific shifts. Continued investigation with larger cohorts and longer storage durations will be important to confirm these observations and to determine whether low‐abundance or more sensitive taxa might still be affected under certain conditions.

The total number of detected bacterial species across all three groups from our study was 473. The highest number of bacterial species was found in Group A (*n* = 174), compared with Groups B and C, which had 153 and 146 species, respectively. Ninety‐eight bacterial genera were not detected across all three storage conditions, with most of the undetected bacteria being anaerobic. Data from this study show that the detection of anaerobic bacteria appears to be influenced by freezing and the length of storage. Ahannach et al. (2021) found that the microbial community of vaginal swabs showed an almost identical taxonomic composition across all storage conditions for each participant, but there were some variations in the taxonomic metagenomics‐based microbiome composition, depending on which species the sample contained. Moreover, they identified a proportionate prevalence of a crucial genus within the vaginal environment (*Gardnerella*) that noticeably increased in certain samples following prolonged storage.

In our study, the abundant anaerobic bacterial genera that we could not detect across the three groups were *Anaerococcus, Prevotella*, and *Peptoniphilus*. This indicates that temperature and storage conditions can influence the detection of anaerobic bacteria. Data from IQC (Figure 1) indicate that the use of the MolYsis DNA kit may contribute to the removal of microbial DNA, potentially impacting the accuracy of microbiome data. IQC strains were diluted in saline water rather than enriched media, which could contribute to the harsh effects of the human depletion step on bacteria. This process may damage the bacterial cell wall, leading to the removal of bacterial DNA. Although our IQC provided some insight into the potential influence of host DNA depletion on microbial representation, our study did not include a direct comparison between depleted and nondepleted clinical samples. Future studies should address this gap to determine the specific effects of HD on cervicovaginal microbiota, particularly in relation to microbial loss or compositional shifts introduced by different depletion methods.

The IQC samples allowed us to assess the technical precision of the extraction and sequencing process. Species spiked at high concentration, such as *E. coli* and *K. pneumoniae*, showed low variability across duplicates, confirming high precision in abundance estimation at adequate read depths (Figure 1). Conversely, species present at lower concentrations, including *Clostridioides difficile* and *F. necrophorum*, displayed more variation in relative abundance between replicates (Figure 1). This is expected, as low‐abundance taxa are more sensitive to stochastic effects and classification thresholds due to fewer reads mapping to species‐specific markers. These findings reinforce the importance of interpreting rare taxa with caution in metagenomic studies and highlight the value of including spiked‐in controls to assess reproducibility.

Freezing and low concentration of glycerol can affect bacterial cell walls, causing the bacteria to lyse and release their DNA. The free microbial DNA is at risk of being removed during the HD step of the purification process using MolYsis Complete5 DNA extraction kit (Molzym GmbH & Co. KG 2024). The effectiveness of the HD process significantly impacts the removal or retention of microbial DNA, thereby influencing the quality and accuracy of microbiome results (Hu et al. 2018). While we found no statistically significant differences in alpha or beta diversity across storage conditions (Figures 2 and 4), small changes in the relative abundance of specific taxa were observed in the IQC samples (Figure 1). Notably, *P. aeruginosa* and *F. necrophorum* appeared reduced at −20°C, whereas *K. pneumoniae* increased. These shifts may reflect species‐specific stability or differential susceptibility to degradation or freeze‐induced lysis, but are also likely influenced by the lower input abundance of some taxa and the inherent variability of detecting low‐abundance organisms. Given that these changes were not consistent across all replicates and did not impact overall diversity patterns, we interpret them cautiously and suggest that future studies consider higher spike‐in replication to quantify these effects more precisely.

A previous study by Poulsen et al. (2021) demonstrated that storage conditions significantly and systematically influenced the taxonomical and functional composition of metagenomics‐based microbiome analyses. They investigated different pig fecal and sewage samples, unspiked and spiked with a synthetic mock community composed of eight microorganisms that included two eukaryotes (*Propionibacterium freudenreichii, Bacteroides fragilis, S. aureus, Fusobacterium nucleatum, E. coli, Salmonella enterica serovar Typhimurium, Cryptosporidium parvum*, and *Saccharomyces cerevisiae*). They showed that frozen storage conditions (−20°C and −80°C for 12 months), had a significant and systematic effect on the taxonomic and functional composition of metagenomics‐based microbiomes, compared with samples that were not stored but processed immediately (0 h). Although the overall community composition appeared stable across storage conditions, species‐level variation was observed in the IQC samples (Figure 1). Notably, *P. aeruginosa* and *F. necrophorum* showed reduced relative abundance after storage at −20°C and −80°C, while *K. pneumoniae*, another Gram‐negative bacterium, remained stable. This suggests that the impact of freezing may be species‐specific and could involve factors such as cell wall integrity, genome accessibility, or DNA fragmentation sensitivity. We did not observe consistent shifts in *Prevotella* or *Mobiluncus* species, both Gram‐negative and relevant to the vaginal microbiome in the main samples. However, given the variability seen in low‐abundance spiked species, we acknowledge that freeze sensitivity of specific taxa cannot be ruled out, particularly for organisms near the detection threshold. This underscores the value of including technical controls and diverse taxa when evaluating sample stability in microbiome studies.

There are also other factors that influenced the microbiome results, such as the sample type, sample collection procedure, transportation, and the DNA extraction method, confirmed by Allaband et al. (2019). These factors could also have played a role in our study. Previous studies indicated that the DNA extraction method and sample collection methods could influence microbiome results (Mirsepasi et al. 2014). The MolYsis Complete5 DNA extraction method used in this study includes a step to remove extracellular DNA, including human DNA released from lysed cells. While this enhances microbial signal by reducing host contamination, it may also introduce bias if certain bacteria lyse during sample storage. In such cases, microbial DNA from fragile or freeze‐sensitive species could be lost during the host depletion step. This mechanism could help explain why some Gram‐negative or anaerobic species, such as *F. necrophorum* or *Prevotella*, showed reduced abundance or inconsistent detection. Although we did not observe significant differences in overall community composition, the selective loss of lysed microbial DNA may confound interpretations at the species level. This is consistent with prior work showing that DNA extraction and storage methods significantly influence microbial community profiles (Fouhy et al. 2015; Dominianni et al. 2014). Future protocols may benefit from DNA extraction methods that minimize extracellular DNA bias, or from preassessment of bacterial integrity post‐thaw to ensure consistent species detection.

Ahannach et al. (2021) investigated the impact of microbial enrichment in samples from different body sites (vagina, skin, and saliva) using two different swab kits (eNAT or MSwab). DNA extraction was performed within 3 h after sampling to evaluate microbial enrichment with three different microbial extraction and host DNA depletion methods (QIAamp PowerFecal DNA kit, QIAamp Microbiome kit, and HostZERO Microbial DNA Kit) compared with no host DNA depletion (QIAamp PowerFecal DNA kit). Without host DNA depletion, the highest concentration of human DNA was found in vaginal samples, followed by saliva and skin for both tested swabs. Without host DNA depletion, saliva samples contained the highest concentration of bacterial DNA, followed by vaginal and skin samples. The large amount of human DNA interferes with the analysis of bacterial content was confirmed by Longhi et al. (2023). Their study showed there was a risk of losing microbial DNA when using a host DNA depletion method of 2.5 wt%/vol saponin. Shotgun metagenomic sequencing revealed inaccurate microbial profiles in the microbial DNA samples, indicating an erroneous increase in Gram‐positive DNA and a reduction of Gram‐negative bacterial DNA. In this study, we used the MolYsis Complete5 DNA extraction kit and Copan Liquid Amies Elution Swab, which could have influenced the quality of microbiome data. This can also be confirmed by the IQC data (Figure 1), which shows that the abundance values of the Gram‐negative bacteria *E. coli* and *P. aeruginosa* are affected. This may explain why we missed some bacterial strains across the three different groups. The effectiveness of the HD process plays a critical role in shaping microbiome profiles, particularly in sample types with high host DNA content such as vaginal swabs. In our study, we used the MolYsis Complete5 DNA extraction kit, which includes a chemical and enzymatic pretreatment step to remove host DNA before microbial lysis. While this method enhances microbial detection by reducing host background, it may also introduce artifacts. Specifically, microbial cells that are already damaged due to freezing or transport may lyse prematurely, causing microbial DNA to be inadvertently removed during the host DNA depletion step. Our IQC results (Figure 1) support this concern: We observed reduced recovery of specific taxa, notably Gram‐negative bacteria, such as *E. coli* and *P. aeruginosa*, which may be more susceptible to cell wall disruption. This aligns with findings from Longhi et al. (2023), who reported a loss of Gram‐negative bacteria following saponin‐based depletion. Thus, while HD improves microbial signal by lowering human DNA, it also introduces a potential bias, especially in frozen or fragile samples. Future protocols should consider these effects, and where feasible, balance host depletion efficiency against the risk of microbial DNA loss.

While overall species richness and diversity metrics remained stable across freeze conditions, species‐level variation was observed (Figures 2 and 4), particularly in low‐abundance Gram‐negative taxa in the IQC samples (Figure 1). These findings suggest that microbial composition may be influenced by storage‐induced lysis or differential DNA stability. Although these taxa were not prominent in our clinical samples, this limitation is important to consider in other metagenomic contexts, particularly when interpreting the abundance of taxa with proposed roles in disease etiology. Researchers should exercise caution when interpreting results involving fragile or low‐abundance bacteria, especially when working with archived or variably stored samples.

Comparing trimmed total reads from microbiome analyses can provide insights into the depth of sequencing performed for each sample. Total reads refer to the number of sequences obtained from a sequencing run, depending on the platform and protocol. This comparison requires careful consideration of experimental design, data quality, statistical analysis, and biological interpretation to ensure meaningful and reliable conclusions. To obtain reliable results, it is crucial to have a sufficient number of reads for microbiome analysis (Allaband et al. 2019). The mean number of our detected trimmed reads from the three groups in our study was 945,430. Zargari Marandi et al. (2022) investigated and considered a threshold for the number of reads must be > 1 million, and that this is necessary for reliable analysis and to provide context and credibility of microbiome results. Usyk et al. (2023) evaluated shotgun read depth, and each genus correlation was quantified at different shotgun subsampling thresholds ranging from 10,000 to 750,000 reads compared with the 16SV4 analyses. They proved that increasing shotgun sequencing depth had a positive effect on correlation with amplicon data, and they recommend sequencing to a depth of over 500,000 reads. This supports that our trimmed reads with mean 945,430 were sufficient.

The majority of diversity analyses indicated no significant impact of storage conditions on vaginal microbiota profiles (Figures 2 and 4). Both Shannon and Simpson indices showed no differences in microbial richness or evenness, and ANOSIM analysis on Bray–Curtis and Jaccard distances did not support clustering by freeze condition. These results suggest that short‐term refrigeration and longer‐term freezing (3 weeks) at −20°C or −80°C do not substantially alter the overall composition or diversity of vaginal bacterial communities. However, an ANOVA‐based PERMANOVA approximation on Bray–Curtis distances (Figure 4) showed a trend toward significance (*p* = 0.061), which may point to subtle changes in community structure. This discrepancy might reflect the greater sensitivity of certain beta diversity metrics or minor technical variations in sample handling.

These findings are consistent with previous research showing that microbial profiles remain relatively stable under common storage conditions. For instance, Song et al. (2016) found that microbiome profiles from fecal samples were largely unaffected by freezing at −20°C or −80°C for up to 6 months. Similarly, Lauber et al. (2010) and Choo et al. (2015) reported negligible differences in microbial community composition across various storage temperatures in skin and saliva samples, respectively. In the context of vaginal microbiota, Forney et al. (2010) emphasized the importance of minimizing preanalytical variation yet found that core bacterial groups are resilient to short‐term handling differences.

Collectively, the results suggest that standard sample handling protocols, including short‐term refrigeration and long‐term freezing, preserve the integrity of vaginal microbiome data, supporting the reliability of biobanked clinical specimens in microbiome research.

### 4.1 Strengths and Limitations

A strength of this study is the inclusion of multiple temperature conditions, providing a comprehensive assessment of common storage practices in microbiome research. The use of IQC was also a strength, as it allowed us to account for the effect of HD on the relative abundance of microbial DNA. A key strength of the study is the use of a well‐defined IQC, which enabled systematic assessment of potential biases introduced by freezing and HD, thereby enhancing the credibility of the microbiome findings. However, a limitation of this study is that the clinical vaginal swab samples were not processed in technical replicates. While this reflects typical clinical practice and allows broader coverage across freeze conditions, it limits the ability to differentiate biological effects from technical variability introduced during DNA extraction, library preparation, and metagenomic classification. To partially mitigate this, we included duplicate IQC samples based on a defined bacterial community, enabling us to estimate the range of experimental variability. Nonetheless, future studies would benefit from technical replication of biological samples, particularly to better resolve subtle changes in low‐abundance taxa. Additionally, the study focused solely on short‐term storage, leaving the effects of longer storage durations unexplored.

## 5 Conclusion

This study evaluated how short‐term storage conditions affect vaginal microbiome composition following HD. Across global diversity metrics, including Shannon, Simpson, Bray–Curtis, and Jaccard indices and relative abundances of the top 10 species, no statistically significant differences were observed across groups. ANOSIM confirmed a lack of group separation (*R* = − 0.042, *p* = 0.937), and a global Kruskal–Wallis test on top taxa supported these findings (*p* = 0.786). Although an ANOVA‐based approximation of PERMANOVA showed a trend toward significance (*F* = 3.51, *p* = 0.061), ANCOM‐BC2 did not identify any taxa as significantly different between conditions. These findings suggest that while overall community composition remains stable under short‐term freezing, subtle shifts in low‐abundance or fragile taxa cannot be ruled out and should be carefully considered in high‐resolution or clinical microbiome studies.

## Author Contributions

**Khaled Saoud Ali Ghathian:** methodology, validation, visualization, writing – review and editing, writing – original draft, formal analysis, project administration, data curation, supervision, resources. **Julie Elm Heintz:** methodology, writing – review and editing, resources. **Sarah Mollerup:** validation, writing – review and editing, resources, formal analysis. **Sarah Juel Paulsen:** methodology, supervision, resources, writing – review and editing. **Karen Angeliki Krogfelt:** writing – review and editing, supervision, project administration, resources, methodology. **Niels Frimodt‐Møller:** methodology, writing – review and editing, resources, supervision, project administration. **Katrine Hartung Hansen:** supervision, writing – review and editing, resources, project administration. **Sofie Ingdam Halkjær:** writing – review and editing, supervision, project administration, resources. **Anne Holm:** resources, writing – review and editing, supervision. **Mette Pinholt:** resources, supervision, methodology, writing – review and editing. **Andreas Munk Petersen:** methodology, writing – review and editing, resources, supervision, project administration, validation.

## Ethics Statement

The samples were collected in relation to a clinical study conducted at Copenhagen University Hospital Hvidovre, Denmark. The study was approved by the Danish Data Protection Agency (P‐2022‐356), and permission for human experiments and recruitment of participants was obtained from the Scientific Ethics Committee for Copenhagen Regional Hospitals, Denmark (Permission no. H‐22014666). The study was performed in accordance with the Revised Declaration of Helsinki. The study was registered at www.clinicaltrials.gov as NCT05553652. All participants provided written informed consent to participate after verbal and written information was given.

## Consent

All participants involved in this study have provided written consent for their data to be published in this article. The consent included approval for the use of anonymized or identifiable data, as applicable, and participants were informed about the nature of the publication and its potential reach.

## Conflicts of Interest

The authors declare no conflicts of interest.

## Supporting information


**Supplementary Figure 1:** The total trimmed reads obtained from the vaginal swabs in the three different groups; A (5°C, 48 h), B (−20°C, 3 weeks) and C (−80°C, 3 weeks).

**Supplementary Figure 2:** Bacterial reads of vaginal microbiome samples in the three groups; A (5°C, 48 h), B (−20°C, 3 weeks) and C (−80°C, 3 weeks).

**Supplementary Figure 3:** The relative abundance (%) of the detected bacterial species in vaginal swabs in the three groups; A (5°C, 48 h), B (−20°C, 3 weeks) and C (−80°C, 3 weeks).

## Data Availability

The microbial metagenomic sequencing data generated in this study, including both vaginal swab samples and IQC samples, have been deposited in the Zenodo repository. The data sets have been processed to remove host (human) DNA in accordance with ethical and legal guidelines under Danish law (Ethical Approval ID: H‐22014666). The data set includes taxonomic profiles (MetaPhlAn4 output), sample metadata, and accompanying quality control data. The data set is publicly accessible via Zenodo at https://zenodo.org/records/15367364. The data that support the findings of this study are openly available in Zenodo at https://zenodo.org/records/15367364, reference number DOI 10.5281/zenodo.15367363.
