# Supplementary material for: Freezing of Vaginal Swabs Prior to DNA Purification Does Not Statistically Significantly Affect Microbiome Composition
Source: Microbiologyopen. 2025 Aug 28;14(5):e70053. doi: 10.1002/mbo3.70053 (PMC12394732; doi:10.1002/mbo3.70053)
Supplement: Supplementary file 2 — Supplementary Figure 2: Bacterial reads of vaginal microbiome samples in the three groups; A (5°C, 48 h), B (−20°C, 3 weeks) and C (−80°C, 3 weeks). [file MBO3-14-e70053-s001.pdf]

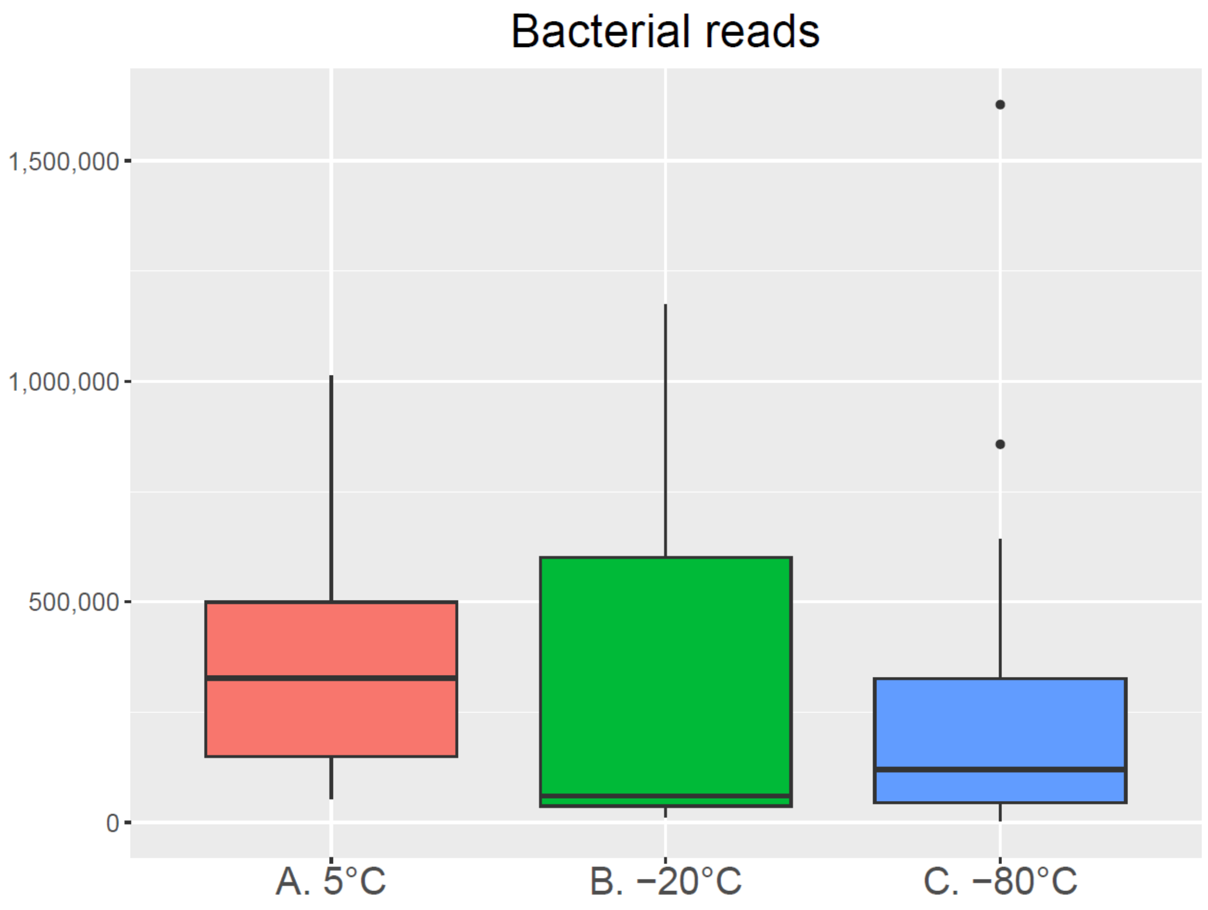

Supplementary figure 2: Bacterial reads of vaginal microbiome samples in the three groups; A (5°C, 48 h), B (– 20°C, 3 weeks) and C (– 80°C, 3 weeks).
