# Supplementary material for: Freezing of Vaginal Swabs Prior to DNA Purification Does Not Statistically Significantly Affect Microbiome Composition
Source: Microbiologyopen. 2025 Aug 28;14(5):e70053. doi: 10.1002/mbo3.70053 (PMC12394732; doi:10.1002/mbo3.70053)
Supplement: Supplementary file 3 — Supplementary Figure 3: The relative abundance (%) of the detected bacterial species in vaginal swabs in the three groups; A (5°C, 48 h), B (−20°C, 3 weeks) and C (−80°C, 3 weeks). [file MBO3-14-e70053-s002.pdf]

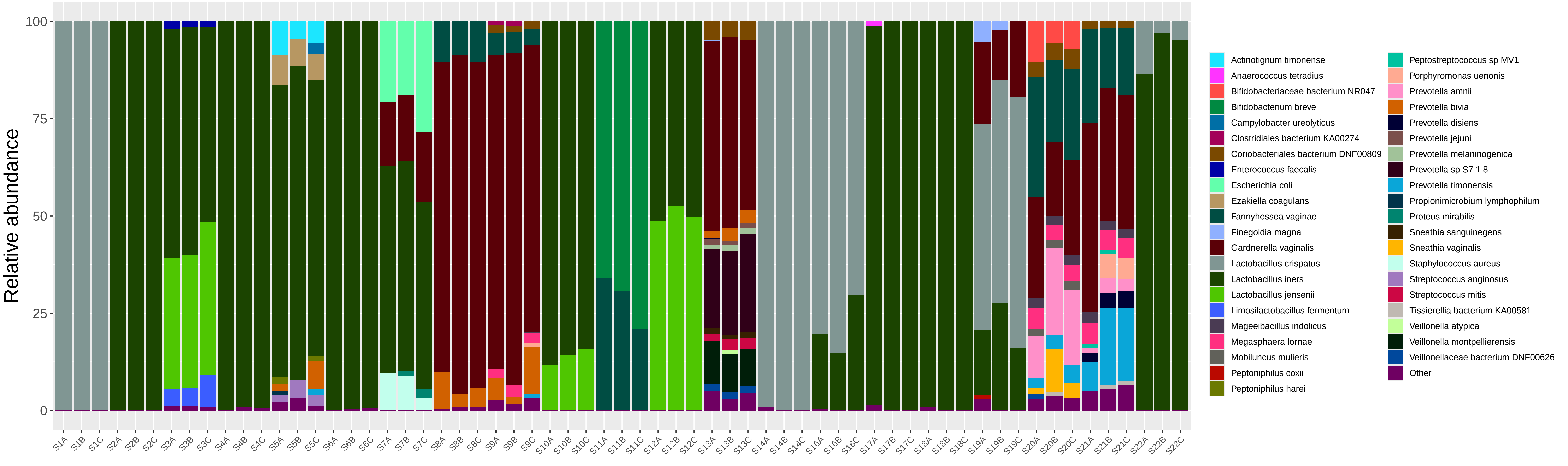
Relative abundance
100
75
50
25
0
S1A S1B S1C S2A S2B S2C S3A S3B S3C S4A S4B S4C S5A S5B S5C S6A S6B S6C S7A S7B S7C S8A S8B S8C S9A S9B S9C S10A S10B S10C S11A S11B S11C S12A S12B S12C S13A S13B S13C S14A S14B S14C S16A S16B S16C S17A S17B S17C S18A S18B S18C S19A S19B S19C S20A S20B S20C S21A S21B S21C S22A S22B S22C
Actinotignum timonense
Anaerococcus tetradius
Bifidobacteriaceae bacterium NR047
Bifidobacterium breve
Campylobacter ureolyticus
Clostridiales bacterium KA00274
Coriobacteriales bacterium DNF00809
Enterococcus faecalis
Escherichia coli
Ezakiella coagulans
Fannyhessea vaginae
Finegoldia magna
Gardnerella vaginalis
Lactobacillus crispatus
Lactobacillus iners
Lactobacillus jensenii
Limosilactobacillus fermentum
Mageeibacillus indolicus
Megasphaera lornae
Mobiluncus mulieris
Peptoniphilus coxii
Peptoniphilus harei
Peptostreptococcus sp MV1
Porphyromonas uenonis
Prevotella amnii
Prevotella bivia
Prevotella disiens
Prevotella jejuni
Prevotella melaninogenica
Prevotella sp S7 1 8
Prevotella timonensis
Propionimicrobium lymphophilum
Proteus mirabilis
Sneathia sanguinegens
Sneathia vaginalis
Staphylococcus aureus
Streptococcus anginosus
Streptococcus mitis
Tissierella bacterium KA00581
Veillonella atypica
Veillonella montpellierensis
Veillonellaceae bacterium DNF00626
Other
